# Bacteria and Butter-Making

**Published:** 1903-03-28

**Authors:** 


### BACTERIA AND BUTTER-MAKING.

Butter-makers, so Mr. W. Marston informs us, are on excellent terms with the bacteria, and have been profiting considerably by the products with which the microbes have supplied them. Cream is rarely churned while fresh; there are exceptions, but in the majority of cases it is subjected first to a process known as ripening or souring. In this method it is allowed to stand in a vat for a time varying from twelve hours to three days, during which interval the bacteria which it contains have an opportunity to multiply. When the ripening is complete, and the micro-organisms in the cream have become very numerous, the churning is done, and after this, the services of the bacteria being no longer required, they are forgotten. Some of them remain in the butter where for the most part they soon die, the rest are washed away in the butter-milk. Those which survive are the agents by which butter subsequently may become rancid. The reason for the ripening of cream is to render it better for butter-making; it churns more rapidly and yields more butter than it does when fresh. But the principal object is to develop the nice flavour characteristic of the highest product, and this good flavour is developed during the microbic process of souring. Whether good or bad tasting butter is obtained depends upon the various kinds of micro-organisms which are present in the cream. Attention is now being directed towards the artificial culture of the most favourable species of bacteria, with a view to obtaining in the future the best flavoured butter alone.

